# A model for irritable bowel syndrome and anxiety comorbidities in relation to alcohol use disorders

**DOI:** 10.3389/fmed.2023.1161130

**Published:** 2023-05-24

**Authors:** Katsiaryna Vashkevich, Kathryn Janiuk, Nasim Maleki

**Affiliations:** ^1^Department of Psychiatry, Massachusetts General Hospital and Harvard Medical School, Boston, MA, United States; ^2^Psychology Research Service, VA Healthcare System, Boston, MA, United States

**Keywords:** chronic pain, alcohol use disorder (AUD), irritable bowel syndrome (IBS), anxiety, model, comorbidity

## Abstract

About 95% of human body serotonin synthesis occurs in the gastrointestinal tract (GI). Lack of sufficient serotonin levels is thought to play a key role in mood disorders, including anxiety disorders. In this study, we looked at a disorder affecting the GI tract, irritable bowel syndrome (IBS), and aimed to determine whether IBS is differentially associated with anxiety disorders in 252 chronic pain patients in the presence of a history of alcohol use disorders (AUD) given that alcohol is an extremely aggressive substance for the GI mucosa. We found that while the prevalence of IBS was not affected by the presence of AUD in chronic pain patients, IBS had significantly higher comorbidity with anxiety disorders in chronic pain patients with comorbid alcohol use disorders. We argue that these findings highlight mechanistic differences in the comorbidity of anxiety disorders with chronic pain and AUD, implicating a central role for GI problems stemming from chronic alcohol use. The findings may have important implications for the treatment of IBS patients with AUD who commonly present with anxiety disorders which could motivate the continuation of problematic drinking and impede recovery success. We propose that addressing GI problems in patients with AUD may help manage AUD and recovery more effectively.

## 1. Introduction

Alcohol, as one of the most abundant and readily available drugs, is a highly potent substance with significant effects on the physiological and cellular level functions, creating fast positive physiological reinforcement due to its decadent effect (1). It has been shown that individuals who live with Alcohol Use Disorders (AUDs) are more likely to also live with an anxiety disorder (2). One study by Boscholoo et al. (3) showed that out of a sample of 1,369 individuals, all individuals at the time of the study had anxiety or depression. The findings further showed that if an individual had remitted or was currently dependent on alcohol, they were 62 or 67% (respectively) more likely than those with no lifetime alcohol use disorder to have an anxiety or depressive disorder at follow-up 2 years later (3). It has been suggested through the self-medication hypothesis that alcohol is used to help individuals cope with anxiety symptoms (4). It has also been shown that individuals with higher rates of anxiety when stopping drinking have a higher risk of slipping than those with lower rates of anxiety when stopping drinking (5). Finally, in a review of studies examining the relationship between stress reactivity and alcohol use and abuse (6), it was concluded that individuals with alcohol dependence and those at risk for alcohol use disorder show different stress responses from comparison control groups whether the stressors are physical, psychological or pharmacological.

Somatically, anxiety appears as an increased heart rate, faster breathing, increased GI tract motility, sweating, sleep deprivation, and many other symptoms. This is primarily due to the activation of the sympathetic nervous system initiated by the amygdala (7), which sends a distress signal to the central nervous system, where the hypothalamus and amygdala communicate with each other and the rest of the body through the autonomic nervous system to generate the appropriate response and reaction. Psychologically, anxiety appears as a feeling of persistent worrying, feelings of danger, fear or fears, feelings of restlessness, inability to focus, anger, or irritability. All these psychological and physiological effects and reactions are usually stopped with alcohol intake. Alcohol consumption slows down the signal transmission in the sympathetic ganglia. It affects the neurotransmitters (dopamine and serotonin), thus creating a positive reinforcement due to the relaxing effects of the first stage of alcohol intoxication (euphoria, impaired critical judgment, and impaired memory) (8). Serotonin is one neurotransmitter that plays a crucial role in modulating anxiety and fear responses and responses to stressful stimuli (9). The amygdala, a key area in the anxiety neurocircuitry, is predominantly modulated by serotonin in responding to stressful stimuli (9). There is a wide range of anxiety-related clinical symptoms, which are usually comorbid with each other, and substance use disorders (10).

Additionally, animal studies show that psychosocial stressors can induce visceral hyperalgesia, activate the hypothalamic–pituitary–adrenal axis and alter mitochondrial activity within the GI tract (11). Additional studies have also differentiated IBS into multiple different types (12). Moreover, studies have shown that patients with IBS constipation type were lower than those without IBS (10). Additional review articles have supported a decrease in the 5-HT transporter (SERT) playing a role in the pathology of IBS (13). Finally, additional patient studies have shown a decrease in 5-HT in the colon of IBS patients (14). In ulcerative colitis patients, there was a decrease in EC cells that correlated to the decrease in 5-HT but not in IBS patients (14). However, the amount of SERT mRNA protein was decreased in IBS (14). It has also been repeatedly shown that gut microbiota changes impact the behavior and functioning of the central and peripheral nervous system, termed the “gut-brain axis” (15). Additionally, animal studies show that psychosocial stressors can induce visceral hyperalgesia, activate the hypothalamic–pituitary–adrenal axis and alter mitochondrial activity within the GI tract (11).

Irritable Bowel Syndrome (IBS) is one of the most common functional GI disorders (16, 17). The pathophysiology of IBS is complex and not well understood yet. However, it is thought to be a multifactorial condition with multiple factors playing a central role in the pathophysiology of IBS (17), including a putative role for abnormalities involving serotonergic signaling mechanisms (14, 18, 19). IBS is often represented as a disorder of dysregulated gut-brain interaction because of the communication between the central nervous system and the gut microbiota that control the GI tract functions (20, 21). IBS could be comorbid with anxiety and depression (22) and other disorders such as sleep and chronic pain (23, 24). Furthermore, chronic stress disorders are thought to have a role in the pathophysiology of IBS (25). Several studies have supported a role for the decrease in the 5-HT transporter (SERT) in the pathology of IBS (13) with one study showing a decrease in 5-HT or serotonin levels in the rectal biopsy specimens taken of the colon of IBS patients (14).

Alcohol is a strong cellular toxin in any amount that disrupts mitochondrial energy processes. It is a highly aggressive substance for the GI tract mucosa (26). The toxicity stems from direct physical effects (drying effect on mucosa), toxic physiological effects (toxic effect on endocrine cells, disruption of the cell membranes), and proinflammatory effects (27). As such, alcohol may disrupt serotonin production in the GI tract through the damage it could cause to the enterochromaffin cells that handle the majority of serotonin synthesis in the body (28). While examining patients’ charts, we noticed a pattern of higher Inflammatory bowel syndrome incidence in patients with chronic alcohol use disorder with anxiety comorbidity and chronic pain. Because of the enteroendocrine cells distributed throughout the gastrointestinal tract and the pathophysiological consequence of long-term combined aggressors’ effects such as alcohol and stress, in this study, we aimed to systematically examine whether a history of alcohol abuse affects the prevalence of IBS and anxiety in chronic pain patients. We made this choice because chronic pain and anxiety are often comorbid, and we were interested in examining the impact of a history of alcohol abuse on the incidence of IBS and anxiety in a patient population with a high prevalence of anxiety disorders. Indeed, anxiety disorders are also highly comorbid with chronic pain disorders and can significantly affect pain perception (29).

## 2. Methods

### 2.1. Sample

The Institutional Review Boards of the Massachusetts General Hospital approved the research. The sample for this study was chosen from the patients evaluated at the Mass General Brigham, consisting of 16 member institutions ranging from academic medical centers and specialty hospitals to community and urgent care centers. As the first step, the International Classification of Diseases (ICD9 and ICD10) diagnostic codes used for claims submitted for billing purposes were used to identify cohorts of interest that were: (1) AUD and Chronic Pain (AUD + CP), and (2) Chronic Pain Only (CP) cohorts. All the ICD9 codes were mapped to ICD10 codes. For AUD, F10.*, and chronic pain, G89.4 and G89.29 ICD10 codes were used. For the AUD + CP cohort, a set of patients with AUD-related codes but no CP-related codes were also identified to capture patients who may have had CP as noted in their clinical notes but had not received a billing code for it. From the patients identified per each cohort, 200 records were chosen randomly by generating 200 indices between 1 and n (where n is the size of the initial cohort identified per the relevant ICD codes) via a random number generator, sorting the cohort by medical record numbers (MRN), and choosing the MRNs corresponding to the randomly chosen indices. This produced a sample of a randomly selected set of 200 patients per cohort with a roughly even distribution across the initially identified lists.

### 2.2. Patient groups

The final patient groups were identified from the sample of patients described above based on an extensive review of the medical charts and the following criteria: The presence of AUD was confirmed if all of the following criteria were satisfied: at least one billing code for AUD existed; there was at least one visit related to alcohol use disorder in the patient’s records; and AUD was mentioned in the clinical notes. The presence of CP was confirmed if all of the following criteria were satisfied: at least one billing code for chronic pain existed, and there was a mention of chronic pain in the clinical notes related to one or more of the following conditions in the chronic form: back pain, neck pain or cervicalgia, joint pain including knee pain, shoulder pain, and hip pain, chronic abdominal pain, arthritis pain, neuropathic pain, fibromyalgia, and cervical myofascial pain. Headaches, including migraine, tension, cluster, or post-traumatic headaches, were not considered chronic pain, and if a patient suffered from chronic headaches only, they were not classified as a CP patient and were omitted. We also excluded cases in which the pain was linked to malignancies.

### 2.3. Anxiety

The presence of anxiety disorders was based on a review of the narrative clinical notes as the primary reference for determination. Anxiety disorders were determined based on persistent complaints (more than 6 months) of feelings of worrying, irritability, fear for the future, fear for health and life, trouble with falling asleep and keeping asleep, difficulties with concentrating, and inability to relax. Patients with situational anxiety or panic attacks were excluded from the cohort. However, the final anxiety group included some patients with panic attacks and situational anxiety since these symptoms were the satellite complaint. We also considered the following ICD10 codes: F41.1, F41.3, F41.8, and F41.9. If there was no mention of anxiety disorder in the clinical notes, but a patient had received one of the noted ICD10 billing codes three or more times, they were labeled as having a history of anxiety disorders.

### 2.4. Irritable bowel syndrome

The presence of IBS was determined by reviewing the clinical notes and examining the associated ICD10 billing codes (K58.*).

### 2.5. Statistical analysis

The difference in the prevalence rates of anxiety disorders between the AUD + CP and CP groups was determined using the chi-square test. Furthermore, separate chi-square tests were performed to examine the differences in the prevalence of IBS in the presence of anxiety in each group.

## 3. Results

### 3.1. Sample

The billing codes included both ICD9 and ICD10 codes. For analysis and reporting, all the ICD9 codes were mapped to ICD10 codes, which will be used to describe the results in the following. Of the cases identified for the AUD + CP group per review of the narrative clinical notes, 97% also had the associated ICD10 billing codes for alcohol use-related disorders (F10. *). As for chronic pain, 93% of the patients also had an associated ICD10 billing code for chronic pain (G89.4 and G89.29).

### 3.2. Patient groups

The final sample consisted of 101 AUD + CP patients (51 men and 50 women) and 151 CP patients (44 men and 107 women). The majority of patients with AUDs were patients who were followed up at our hospitals for a long time, with 84% of the patients in our cohort evaluated more than three times for AUDs. All patients in the AUD + CP cohort were diagnosed with alcohol abuse as determined per review of the charts and further confirmed by the ICD9 and ICD10 codes they had received. Of all the patients, 71% also had received additional ICD9 or ICD10 codes for alcohol dependence, and 46% had experienced periods of being in remission. The majority of the patients with chronic pain had a history of being on prescribed or over-the-counter pain medication for managing their chronic pain. The two groups had no significant age difference (AUD + CP: 55.7 ± 8.4 years, CP: 54.2 ± 9.8 years). History of other substance use disorders (e.g., opioids, cocaine, etc.) was considerably more common in the AUD + CP patients compared to the CP patients (43% vs. 13%).

### 3.3. Anxiety

**Figure 1:**
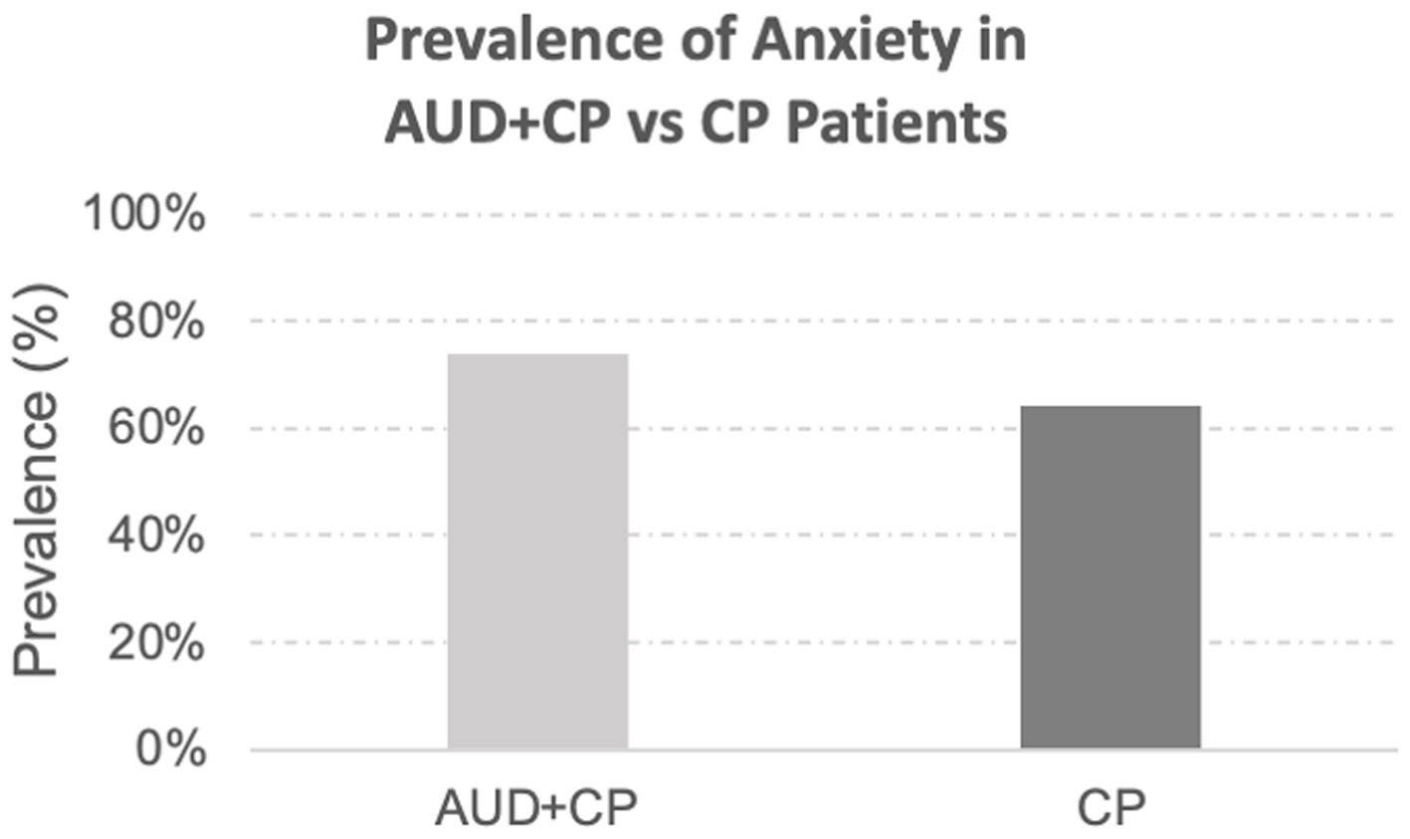
Prevalence of anxiety disorders. The prevalence of anxiety disorders was slightly higher in the AUD + CP group (AUD + CP: 74% vs. CP: 64%), although the difference was not statistically significant.

In the final sample, 29 out of 171 patients identified with anxiety disorders were considered to have a history of anxiety disorders per the associated billing codes only. Of the remaining 171 patients, 19 had no ICD10 billing codes for their anxiety disorders, and determinations were based solely on the narrative clinical notes. The remaining 152 patients had the relevant associated billing codes, and the presence of anxiety disorders was discussed in their narrative clinical notes. There was no difference in the incidence rate of anxiety in the CP vs. AUC + CP groups, Figure 1. In both groups, anxiety was highly comorbid with depression, constituting 94% of the AUD + CP patients. In addition, 79% of the CP patients who suffered from anxiety also suffered from depression.

### 3.4. Irritable bowel syndrome

**Figure 2:**
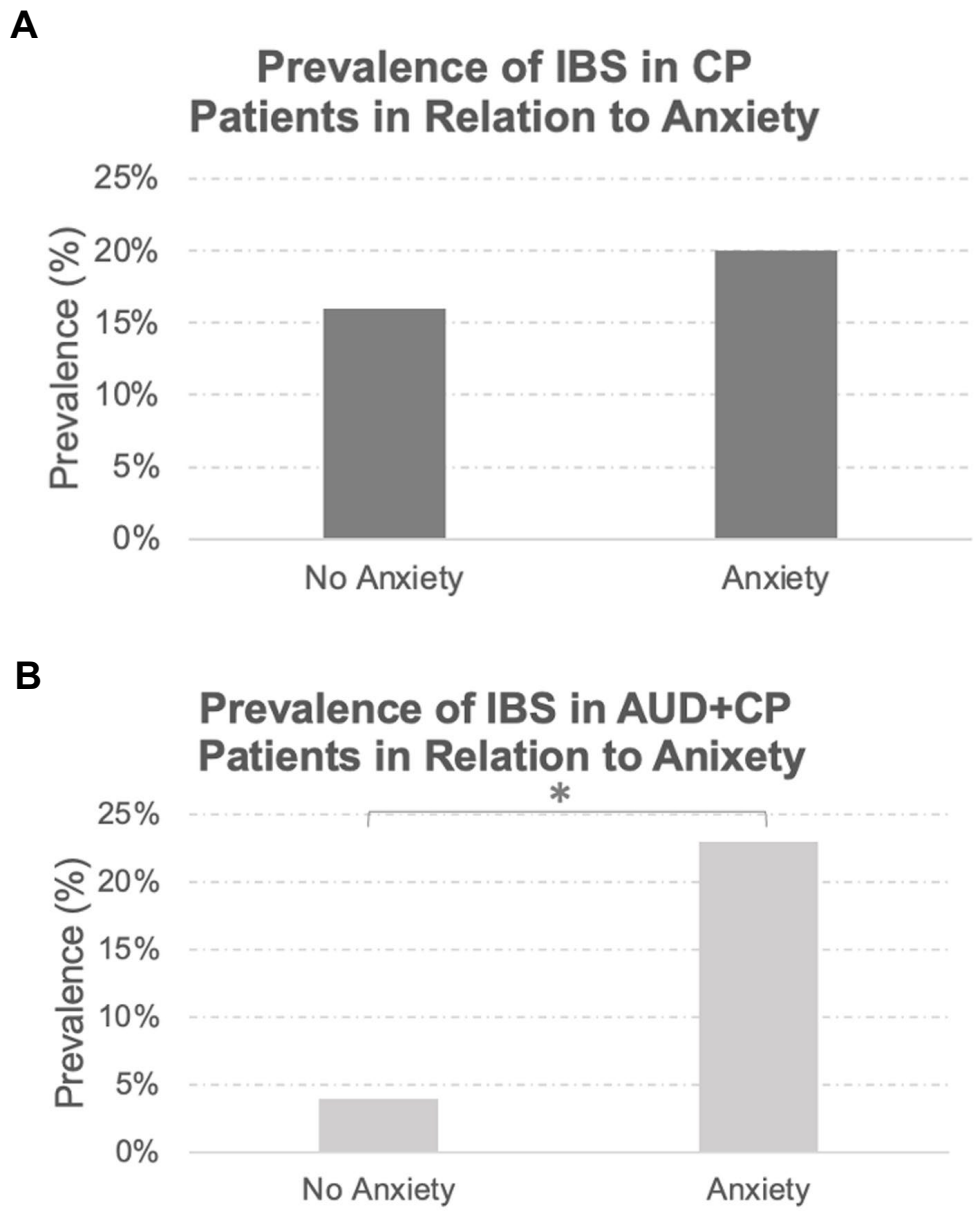
Prevalence of IBS in Relation to Anxiety Disorders. While the prevalence of IBS was similar between the AUD + CP and CP groups (18 and 19%, respectively), the prevalence of IBS was significantly higher in the AUD + CP group in the presence of anxiety disorders **(A)**, where 23% of IBS cases were associated with anxiety disorders. Only 4% were associated with no anxiety disorders. However, the same pattern was not observed in the CP group **(B)**; 20% of IBS cases were associated with anxiety disorders, and 16% were associated with no anxiety disorders. In other words, the prevalence of IBS was not linked to the presence or absence of anxiety in the CP group in any specific direction. However, it was strongly associated with anxiety disorders in AUD + CP patients. *Denotes statistical significance at *p* < 0.001.

**Figure 3:**
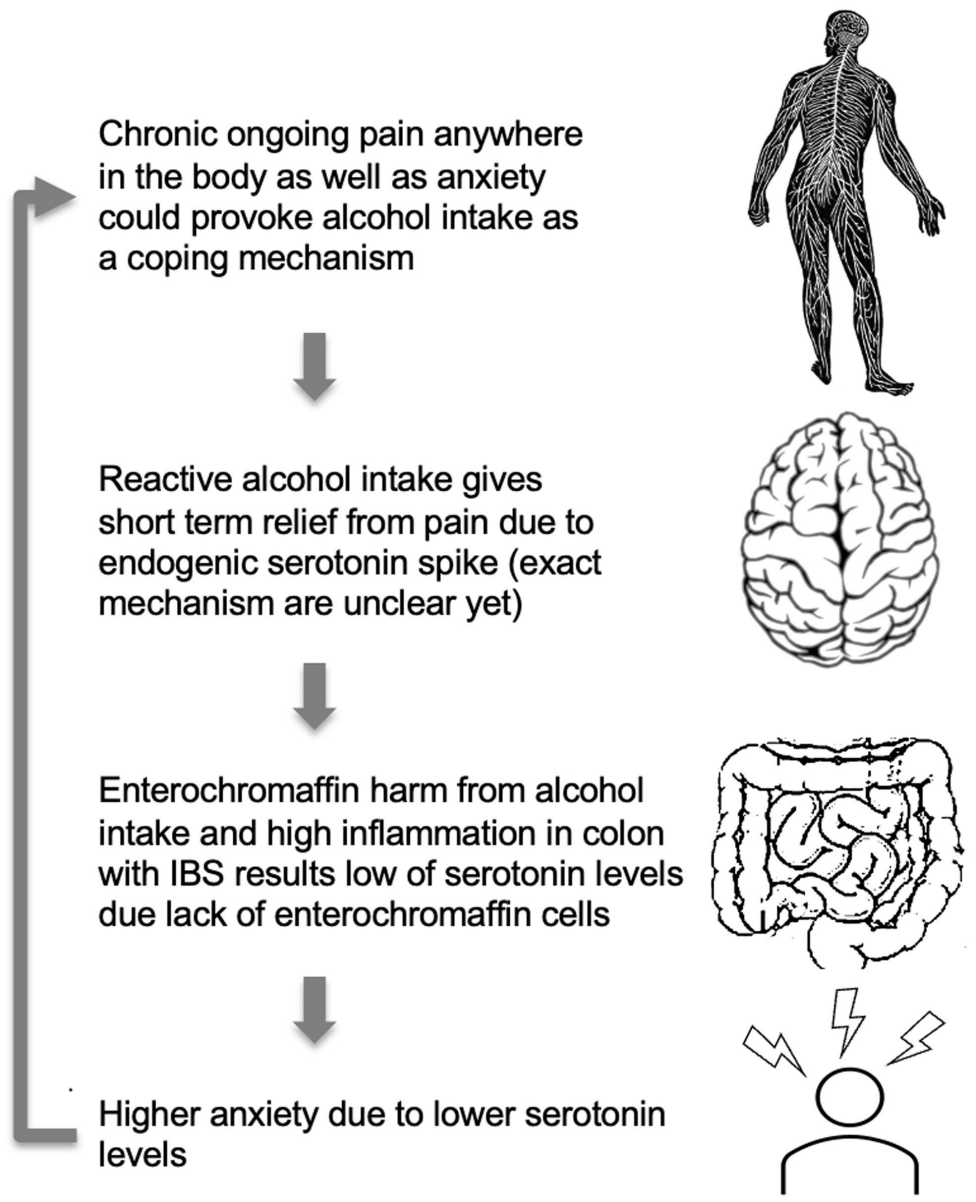
A model for higher anxiety levels in IBS patients with comorbid CP and AUD. High anxiety levels, as well as chronic pain, may provoke alcohol consumption. Alcohol, due to toxic ethanol features, has a suppressing effect on gut microbiota and a direct harmful effect on enterochromaffin cells and serotonin production (66). Alcohol intake may affect gut condition through the direct cytotoxic effect of ethanol and alcohol metabolites on the serotonin-producing enterochromaffin cells leading to low serotonin production. The cycle repeats itself as higher anxiety due to lower serotonin levels would again provoke alcohol intake.

A total of 46 patients in the whole sample had a history of IBS. All these patients also had a history of chronic pain involving different body sites, of whom 88% had reported a history of pain at multiple sites in the body. The prevalence of IBS was similar between the two groups (AUD + CP: 18% vs. CP: 19%), with no statistically significant difference between the groups. The majority of patients with IBS in both groups were women (AUD + CP: 72%, CP: 86%). There was a drastic difference in the incidence of IBS in patients with and without anxiety in the AUC + CP group in that IBS was about three times more common in those with anxiety in the AUC + CP group, Figure 2. No such difference was observed in the CP group, and the incidence of IBS was similar in those with and without anxiety in the CP group, Figure 3. We also compared two other GI tract disorders: Crohn’s disease and ulcerative colitis. However, these disorders were not common in our sample (Crohn’s disease: 1% and Ulcerative Colitis: 4% of the total sample), and there were no differences between the two groups in the incident rate of these conditions. No difference was observed in their prevalence in relation to anxiety.

## 4. Discussion

In this study, we examined the prevalence of IBS and the comorbidity of anxiety with IBS in those who only had chronic pain versus those who also had alcohol use disorders in addition to suffering from chronic pain disorders. We found that while the prevalence of IBS was not affected by the presence of AUDs in chronic pain patients, IBS had significantly higher comorbidity with anxiety disorders in chronic pain patients with comorbid alcohol use disorders. We argue that these findings highlight mechanistic differences in the comorbidity of anxiety disorders with CP and AUD and implicate a central role for GI problems stemming from chronic alcohol use that may underlie the higher comorbidity of anxiety with IBS in CP patients in the presence of AUD. In the following, we will discuss some potential mechanisms that may explain the patterns observed in this study.

### 4.1. Potential role of serotonin

One potential mechanism explaining the observed patterns may be through the interference of chronic alcohol use with the proper functioning of the gut wall (through causing inflammation and chronic stress) that may affect the synthesis of serotonin, which is a critical neurotransmitter in regulating mood and emotion, among other functions via different subtypes of serotonin receptors expressed on CNS structures. In fact, altered serotonin signaling is noted in both IBS and ulcerative colitis, with decreased normal mucosal serotonin secretion (14). Previous research has suggested a link between abnormal functioning of 5-HT and the HPA axis contributing to the visceral sensitivity seen in IBS (30). Alcohol consumption increases the level of serotonin through increased secretion in the brain or slower clearance from the synapse (8). Increased serotonin level leads to euphoria, decreased pain, decreased anxiety and depression, and an overall better mood (31). Patients with underlying stress disorders such as anxiety or depression may find that the consumption of alcohol helps them temporarily diminish the symptoms of their disorder (8).

### 4.2. Damage to the GI mucosa

Chronic and even sporadic accidental alcohol consumption is damaging to GI mucosa. In the upper GI tract, alcohol acts as a carcinogenic factor (32) (Implicated in squamous cell carcinoma of the larynx and adenocarcinoma of the esophagus). In the lower GI tract, it causes alcohol-induced mucosal damage, thus affecting gut microbiota and the gut-brain axis, disrupting normal signaling from GI tract epithelium to the brain via the myenteric plexus and peripheral nervous system (31). Enterochromaffin cells are accessible to gut microbiota and microbiota metabolites on the GI lumen epithelial side and connected with afferent and efferent nerve terminals on the lamina propria of the GI tract wall (33). This bidirectional signal transmission by EC cells (from the GI tract lumen to the brain and back) is not yet completely understood. EC produces 5HT (serotonin precursor) in response to various central and peripheral stimuli, such as stress-induced GI tract hypermotility, aggressors such as alcohol, and microbiota metabolites. After secretion, it is taken by platelets, stored in granules in different tissue, and released upon activation (31). Furthermore, serotonin activates its own receptors on primary afferent nerve terminals in the proximity of enterochromaffin cells (34). This can modulate various gut functions through the activation of enteric reflexes that mediate the autonomic neural control of the GI tract and the activation of the HPA axis and the endogenous pain-modulation systems (34). Activation of these reflexes, in turn, can mediate pain perception and inflammation in the GI tract, among other things (34).

### 4.3. Model

One potential model to explain the observed patterns could be that alcohol dries and disrupts gentle mucosal and enterochromaffin cell membranes in the gut wall and affects colonic epithelial cells’ life cycle leading to disruption of microbiota composition and functioning (35). As such, it is likely that due to chronic alcohol use, altered enterochromaffin cells from colonic mucosa cannot produce the necessary amount of serotonin and naturally support mood. This, in turn, can create a vicious circle where stress and anxiety feeling leads to alcohol consumption with serotonin spike and GI mucosa damage as a result. That, in turn, could lead to low peripheral serotonin secretion and higher anxiety and depression during the hangover and sober period, leading to more alcohol consumption due to the positive reinforcement effect, as depicted in Figure 3.

While this proposed model is simple and not comprehensive in that it does not include other possibilities (such as the involvement of damage to different cell types and the potential consequence of such damages), we have some preliminary data that provide some support for the proposed model: in a separate preliminary sample using medical chart data of 27 adult patients (13 women and 14 men) with a history of alcohol abuse, we found that about 70% also had a diagnosis of anxiety or depressive disorders, of whom more than half (53%) had quite low serum serotonin levels (<50 ng/ml) as determined using a similar test for all of the patients. While this sample size is small, this observation needs to be verified in a much larger sample. While the link between damage to the EC cells in the gut and anxiety in AUD may be more complex than just related to low serotonin levels, the high percentage of patients with both low serotonin levels in our preliminary sample may provide some support for our proposed model for some if not all individuals with AUDs.

### 4.4. Chronic pain

In this research, we primarily focused on patients with chronic pain. Chronic pain is defined as pain with a duration of greater than 3 months (36). There is a high prevalence of anxiety in patients with chronic pain. For instance, a population study in 2003 (37) representative of the US population showed that self-reported pain and disability levels were associated with anxiety disorders. Several studies have suggested the reasons for the comorbidity of anxiety and chronic pain. A study by Narita et al. in 2006 (38) suggested they were potentially comorbid due to potential changes in the function of the μ-opioid receptor located in the amygdala. Another study in 2020 by Jin et al. (39) suggested a novel pathway that projected glutamatergic neurons from layer 5 of the somatosensory cortex to GABAergic neurons of the caudal dorsolateral striatum, which could potentially control some aspects of anxiety in chronic pain. Both studies suggest that the comorbidity of anxiety and chronic pain is due to changes in the brain, whether involving connectivity patterns or receptor function (38, 39).

Pain could also be a reactive response of enterochromaffin cells to irritation caused by environmental or internal triggers. This occurs because enteroendocrine cells have multiple receptors expressed on their surface that enable them to react to such stimuli. One of the pathways is through the 5-HT3 serotonin subtype receptor, which can send nociceptive signals to the spinal cord (40). Serotonin itself participates in regulating not only stress and pleasure but also in regulating the response to noxious stimuli via multiple pathways through nerve endings (41). We propose that due to the cytotoxic effect of alcohol on enterochromaffin cells and serotonin release alteration in the presence of chronic stress such as anxiety, gut mucosa’s reactive response presents in the form of a group of multiple symptoms, including abdominal pain. At the same time, the same factors may provoke more robust pain hypersensitivity in patients with anxiety disorders and AUD. The exact signaling and pathophysiology mechanisms of EC cell alteration by anxiety and alcohol and IBS pathophysiology still needs to be explored.

### 4.5. Other factors

While we interpreted the patterns observed in the context of damage to serotonin-producing ECs in the GI tract, damage to other cell types may also be involved. For instance, chronic alcohol consumption may mediate mast cell activation in the gut to produce inflammatory markers that could irritate or damage other cell types, including the ECs (42). Additionally, increased mast cells are associated with several chronic pain disorders (43). It has been well-documented that an increase in mast cells can lead to central sensitization and chronic pain (44–46). Increased mast cells have also been shown to cause anxiety (47).

Moreover, mast cells may also be implicated in the pathology of IBS (48). However, given the above, if mast cells were potentially causative of the anxiety being seen in AUD patients with IBS, the incidence of anxiety in CP patients should not have been comparable to that of AUD + CP patients, Figure 1, and we would have expected to see more incidence of anxiety in the AUD + CP patients (47, 49, 50). Similarly, involvement or interaction with dopamine, histamine, GABA, and other endocrine hormones, as well as other neurotransmitters that might play a role in IBS, anxiety, or alcohol use disorder and chronic pain, may also play a role in explaining the patterns that we observed (51, 52).

For instance, multiple studies have shown alterations in dopamine level in patients with IBS (lower level of dopamine) and dopamine plays a role in peristalsis (53) as well as in anxiety and pain (54). Furthermore, preexisting conditions and lifestyle are also factors that could affect the GI tract mucosa and anxiety and play a role in the pathophysiology of IBS (55). It is also important to note that mucosal damage due to alcohol use could affect the goblet cells with the consequent generation of dysbiosis and increasing gut permeability allowing gut microbial components into the circulation and enter CNS and potentially causing inflammation which may underlie anxiety (56–58). However, again, given that no difference was observed in the anxiety incidence rates between AUD + CP and CP groups, it is less likely that this mechanism alone could explain the pattern we see in the data. It is also important to note that chronic excessive alcohol use could lead to adaptive changes in the brain, such as reduction in GABA receptors, reduction in dopamine release in the mesolimbic system, and particularly interfere with the balance between excitatory and inhibitory neurotransmitters over time (59) that may play a role in increasing the likelihood of anxiety disorders (60). Nevertheless, given the patterns observed in this study, the role of serotonin signaling alterations could not be denied in this particular combination of comorbidities, such as IBS, in patients with an alcohol disorder, anxiety, and chronic pain (61, 62).

### 4.6. Other findings

There were some other notable patterns in our dataset. First, there were more women in the CP group (107 women versus 44 men), whereas in the AUD + CP group, there was the same number of men and women. These patterns are consistent with the prevalence patterns of chronic pain disorders that predominantly affect women and the prevalence patterns of alcohol use disorders that predominantly affect men (63). The disproportionate prevalence of these conditions in men and women seems to balance out in our sample when both conditions are present. We also noted a higher prevalence of other substance use disorders among AUD + CP patients, consistent with a higher prevalence of other substance use disorders among those with AUDs (64). Finally, patients with chronic pain who also suffer from alcohol use disorders may seem to have a higher level of susceptibility to gastrointestinal tract disorders as well as Inflammatory bowel syndrome, probably due to harming effect of alcohol on colonic mucosa affecting both microbiotas as well as enterochromaffin cells (65). Our data, however, showed no difference in the prevalence of IBS in the AUD + CP group compared to the CP group.

### 4.7. Future directions

Future studies could examine how stress affects the GI tract condition – both mucosal damage and microbiota composition in AUD and explore the mechanisms of interaction between AUD and IBS at a molecular level. Furthermore, future studies can examine how treating underlying mechanisms of GI tract conditions such as IBS may positively affect the mental health disorders such as anxiety and depression as well as chronic pain in those with AUDs.

## Data availability statement

The original contributions presented in the study are included in the article/supplementary material, further inquiries can be directed to the corresponding author.

## Author contributions

KV and NM contributed to the conception and design of the study. KV performed the chart review and data extraction. NM performed the statistical analysis. KV, NM, and KJ wrote the first draft of the manuscript. All authors contributed to the manuscript revision, read, and approved the submitted version.

## Funding

The writing of this manuscript was supported by a grant from the National Institute on Alcohol Abuse and Alcoholism (NIAAA) K01-AA027833.

## Conflict of interest

The authors declare that the research was conducted in the absence of any commercial or financial relationships that could be construed as a potential conflict of interest.

## Publisher’s note

All claims expressed in this article are solely those of the authors and do not necessarily represent those of their affiliated organizations, or those of the publisher, the editors and the reviewers. Any product that may be evaluated in this article, or claim that may be made by its manufacturer, is not guaranteed or endorsed by the publisher.

## Abbreviations

AUD: alcohol use disorder
CP: chronic pain
GI: Gastrointestinal
IBS: irritable bowel syndrome
EC: enterochromaffin cells
